# Analysis of the Alterations in Symbiotic Microbiota and Their Correlation with Intestinal Metabolites in Rainbow Trout (*Oncorhynchus mykiss*) Under Heat Stress Conditions

**DOI:** 10.3390/ani15142017

**Published:** 2025-07-08

**Authors:** Changqing Zhou, Fengyuan Ding

**Affiliations:** 1 Institute of Livestock, Grass, and Green Agriculture, Gansu Academy of Agricultural Sciences, Lanzhou 730020, China; zhoucq@lzu.edu.cn; 2 Gansu Provincial Station of Aquatic Technology and Txtension, Lanzhou 730070, China

**Keywords:** cold-water fish, commensal microbiota, physiological adaptation, metabolic profile

## Abstract

Global warming represents one of the most pressing environmental challenges to cold-water fish farming. Heat stress markedly alters the mucosal symbiotic microbiota and intestinal microbial metabolites in fish, posing substantial barriers to the healthy artificial breeding of rainbow trout (*Oncorhynchus mykiss*). However, the relationship between mucosal commensal microbiota, intestinal metabolites, and host environmental adaptability under heat stress remains poorly understood. In this study, rainbow trout reared at optimal temperature (16 °C) served as controls, while those exposed to maximum tolerated temperature (24 °C, 21 d) comprised the heat stress group. Using 16S rRNA amplicon sequencing and ultra-high-performance liquid chromatography–mass spectrometry (UHPLC-MS), we analysed the mucosal commensal microbiota—including gastrointestinal digesta, gastrointestinal mucosa, skin mucus, and gill mucosa—and intestinal metabolites of rainbow trout under heat stress conditions to explore adaptive and regulatory mechanisms. Analysis of microbial composition and diversity revealed that heat stress exerted the greatest impact on the diversity of gill and skin mucus microbiota, followed by gastrointestinal digesta, with relatively minor effects on the gastrointestinal mucosa. At the phylum level, Proteobacteria, Firmicutes, and Bacteroidetes were predominant in the stomach, intestine, and surface mucosa. At the genus level, *Acinetobacter* showed the greatest increase in abundance in skin and gill mucosa under heat stress, while *Enterobacteriaceae* exhibited the most pronounced increase in intestinal digesta, gastric digesta, and gastric mucosa. Differential metabolites in the intestinal digesta under heat stress were predominantly enriched in pathways associated with amino acid metabolism, particularly tryptophan metabolism. This study provides a comprehensive characterisation of microbiota and metabolic profile alterations in rainbow trout under heat stress condition, offering a theoretical foundation for understanding the response mechanisms of fish commensal microbiota to thermal stress.

## Simple Summary

Rainbow trout is a cold-water fish species that exhibits a stress response when water temperature exceeds 20 °C under artificial rearing conditions. With ongoing global warming, elevated water temperatures have significantly impaired the physiological functions and metabolic activities of cold-water fish. By analysing alterations in the symbiotic microbiota of the mucosa and gastrointestinal digesta in rainbow trout under heat stress conditions, alongside the associated intestinal microbial metabolites, the present study aims to elucidate the physiological adaptation mechanisms of cold-water fish to environmental changes, thereby providing theoretical and technical support for artificial breeding and mitigating production losses.

## 1. Introduction

Heat stress refers to an abnormal physiological state experienced by fish when environmental temperatures exceed their capacity for physiological regulation [1,2,3]. This stress response triggers a series of physiological and behavioural changes that can adversely affect fish health and survival. With the progression of global warming, heat stress has emerged as a major threat to the cold-water fish aquaculture industry. Rainbow trout (*Oncorhynchus mykiss*), a farmed cold-water species, is highly sensitive to fluctuations in water temperature. When water temperatures exceed 20 °C, rainbow trout exhibit a stress response characterised by reduced feed intake, intestinal damage, immunosuppression, decreased disease resistance, and, in severe cases, mortality [1,2,3]. Research has indicated that increased ambient temperatures can diminish both the relative abundance and diversity of gut microbiota across various of animal species, often resulting in negative impacts on host well-being. Such outcomes have been documented in animals including the Chinese giant salamander [4], red-backed salamander [5], common lizard [6], and laying hens [7]. In the case of rainbow trout, elevated temperatures have been linked to a decrease in beneficial lactic acid bacteria and a concurrent rise in Mycoplasma populations within the intestinal contents, which may compromise gut health [8]. However, limited research has examined the effects of elevated water temperatures on the commensal microbiota of fish mucosa, particularly with respect to changes in the skin mucosa, gill mucosa, gastrointestinal mucosa, and gastrointestinal digesta under heat stress. Investigating these changes may provide further insights into the mechanisms underlying the adaptation of host-associated microbiota to environmental temperature variations.

Studies have documented the presence and structure of microbiota across distinct anatomical regions in numerous aquatic species, such as *Penaeus monodon* [9], *Atlantic salmon* [10], *Acipenser baerii* [11], and *Neophocaena asiaeorientalis sunameri* [12]. These studies have demonstrated expected variations in microbial composition and abundance across tissues such as the skin, gills, and intestine, as well as in digestive products including faeces [13]. The environmental adaptability of fish is also closely associated with the abundance and composition of their symbiotic microbiota. Nevertheless, limited information is available regarding the alterations in fish microbiota under heat stress, and the associated mechanisms of adaptation and regulation. In the present study, we examined changes in the composition and structure of microbiota within the intestinal digesta, intestinal mucosa, gastric digesta, gastric mucosa, and surface mucosa (skin and gills), along with the metabolite response patterns in the intestinal digesta of rainbow trout under heat stress condition.

Combining microbiome and metabolome analyses is widely regarded as an effective strategy for exploring interactions between hosts and their microbial communities [14]. Previous studies suggest that the metabolic activity of intestinal microbiota is key to keeping host homeostasis and health, particularly under extreme environmental conditions [15]. Alterations in the intestinal microbiota may lead to changes in intestinal metabolites, subsequently influencing host phenotypes. Based on this, we hypothesised that mucosal commensal microbiota are correlated with the metabolic profile of intestinal digesta in rainbow trout under heat stress condition. Furthermore, the interaction between mucosal microbiota and gut metabolites may contribute to the environmental adaptability of rainbow trout. This study consequently establishes a foundation for further investigation into the mechanisms underlying environmental adaptation in fish. To investigate this, we utilised 16S rRNA gene sequencing alongside untargeted metabolomic profiling via ultra-high-performance liquid chromatography–mass spectrometry (UHPLC-MS), aiming to evaluate how heat stress influences both the gut microbiota and intestinal metabolite composition in rainbow trout.

## 2. Materials and Methods

### 2.1. Fish and Facilities

The rainbow trout were obtained from a salmon and trout breeding base in Linxia, China, and reared in a water recirculation aquaculture system. Healthy, disease-free, and one-year-old full-sibling Norwegian rainbow trout with uniform body length and comparable body weight (average body weight: 162.4 ± 2.7 g) were selected for the experiment. Throughout the trial period, the fish were fed a commercial pelleted diet (crude protein ≥ 48.0%, crude fat ≥ 10.0%) twice daily (08:00 and 17:00). The feed was purchased from Beijing Han-ye Technology Co., Ltd. (Beijing, China). Prior to the experiment, the fish were maintained at 16 °C for a two-week acclimation period. The experimental design included two treatment groups: a control group (CO) and a heat-stressed group (HS). Each group consisted of three biological replicates, with 20 fish allocated to each replicate, resulting in a total of 120 fish distributed evenly across six separate tanks. At the beginning of the experiment, the control group remained at the optimal growth temperature of 16 °C, while the heat stress group was subjected to a high-temperature condition of 24 °C—the maximum the fish could tolerate. This thermal exposure continued for a duration of 21 days. Each experimental unit consisted of a 600 L cylindrical cement tank (100 cm in diameter), outfitted with three 1000 W electric heaters and an automated system to monitor and regulate water temperature consistently. A high-power aeration pump was used throughout the experiment to ensure continuous oxygenation, maintaining dissolved oxygen levels above 7 mg/L. All sampling procedures involving rainbow trout were performed in accordance with the guidelines and regulations of the Animal Ethics Committee and were approved by the Lanzhou University Animal Ethics Committee (EAF2021042).

### 2.2. Sample Collection

Prior to sample collection, fish were anaesthetised using 20 mg/L MS-222 (tricaine methanesulfonate; Argent Chemical Laboratories, Redmond, WA, USA). Tissue samples were then carefully dissected under aseptic conditions using sterile tools on an ultra-clean workbench. Three fish were randomly selected from each tank, resulting in a total of nine fish per group (N = 9) and eighteen fish across both the CO and HS groups. Skin mucus samples were collected by scraping the dorsal and ventral surfaces of each fish with a sterile scalpel. Gill mucosa samples were obtained by randomly excising gill tissue using sterile instruments. Gastrointestinal digestive and mucosal samples were collected from the entire stomach and intestines. It should be clearly noted that ‘digesta’ samples refer to residual incompletely digested food within the gastrointestinal tract. Gastric and intestinal digesta were collected by separately opening the stomach at the pyloric orifice and the intestine at the hindgut (1.0 cm anterior to the anal opening). Digesta from the nine fish in each group were pooled into a shared sterile tube to create a sample mixture pool, and then aliquoted into six sterile Eppendorf tubes. Furthermore, we confirmed that no other factors such as fish tanks had any impact on the experiment results. Gastric and intestinal mucosa were rinsed with phosphate-buffered saline (PBS) prior to collection, then scraped using a sterile scalpel and transferred to sterile Eppendorf tubes for storage.

### 2.3. Microbiota Analyses

The gastrointestinal content sample (approximately 220 g), as well as appropriate amounts of skin mucus, gill mucus, and gastrointestinal mucosa samples, were used for DNA extraction. These samples were placed into a sterile tube containing silica beads and homogenised using a tissue grinder (Bertin Instruments, Montigny-le-Bretonneux, France). Genomic DNA was isolated from the homogenates according to the manufacturer’s protocol provided with the Qiagen DNA extraction kit (Hilden, Germany). The concentration and purity of the resulting DNA were subsequently measured. To amplify the V3–V4 regions of the 16S rRNA gene, we employed the forward primer 338F (sequence: 5′-ACTCCTACGGGAGGCAGCA-3′) and the reverse primer 806R (sequence: 5′-GGACTACHVGGGTWTCTAAT-3′). After PCR amplification, the products were purified, quantified, and combined to create sequencing libraries. High-quality libraries were sequenced on the Illumina NovaSeq 6000 platform (Beijing Baimaike Biotechnology Co., Ltd.; Beijing, China). The raw data obtained through high-throughput sequencing were processed through Base Calling and converted into raw sequence reads in FASTQ format. Quality filtering of the raw reads was performed using Trimmomatic v0.33, followed by paired-end read assembly using Usearch v10 and chimaera removal using UCHIME v4.2, resulting in high-quality effective reads.

Sequence data were processed using the Usearch tool [16], which grouped the reads into taxonomic clusters based on a 97% sequence similarity threshold. Features with very low abundance (below 0.005%) were excluded from downstream analysis. Feature sequences were taxonomically classified using a naïve Bayes algorithm, referencing the SILVA database for accurate assignment. Taxonomic classification was assigned at the phylum, class, order, family, genus, and species levels. Using QIIME, we constructed species abundance profiles across various taxonomic ranks, while visualisation of community structure at each level was performed using tools within the R programming environment. Alpha diversity metrics—such as observed ACE, Chao1, Shannon, and Simpson indices—were computed via QIIME2. Statistical comparisons of alpha diversity across experimental groups were carried out using R software (vegan).

To examine alpha diversity in microbial communities, data normality was first verified, followed by statistical analysis using Student’s *t*-test. For beta diversity, both weighted and unweighted UniFrac distance metrics were applied. Multivariate analyses—including principal component analysis (PCA), principal coordinates analysis (PCoA), and non-metric multidimensional scaling (NMDS)-were conducted based on operational taxonomic unit (OTU)-level data to explore community differences. To identify taxa with significantly different abundances between the CO and HS groups, linear discriminant analysis (LDA) effect size and Metastats were employed, aiding in the detection of potential microbial biomarkers.

### 2.4. Metabolic Profile Analyses of Gut Content

UPLC-MS was used to investigate the intestinal metabolic composition of rainbow trout subjected to thermal stress. Approximately 100 mg of intestinal content was precisely measured and immediately pulverised.

For metabolite extraction, 300 µL of methanol and 20 µL of internal standard were added to 100 µL of homogenised sample, followed by vortexing for 30 s. The mixture underwent ultrasonic treatment for 10 min in an ice-water bath and was subsequently left at –20 °C for 1 h. After centrifugation at 13,000 rpm for 15 min at 4 °C, 200 µL of the resulting supernatant was collected. From each sample, 20 µL was combined to create a quality control (QC) sample, and the remaining 200 µL was reserved for instrumental detection.

An Agilent 1290 Infinity LC UHPLC system was used for chromatographic analysis, outfitted with a BEH Amide column from Waters (1.7 μm, 2.1 × 100 mm), and instrumental settings were based on protocols from Tian et al. [17]. An AB Sciex Triple TOF 6600 mass spectrometer was used, with configuration parameters also derived from Tian et al. [17]. Spectral data were converted to the mzXML format using ProteoWizard (version 3.6.1) and analysed with the XCMS software (version 3.2) for retention time correction, metabolite identification, peak deconvolution, and alignment. The minfrac value was set to 0, and the minimum intensity threshold was set at 0.6.

Metabolite classification was carried out using the LIPID MAPS database (http://www.lipidmaps.org/, accessed on 24 January 2024). Dimensionality reduction and classification were achieved using PCA and OPLS-DA, respectively. Key differential metabolites were selected by evaluating their fold change metrics. Metabolites showing significant differences were identified based on fold change (FC), *t*-test *p*-values, and VIP values derived from the OPLS-DA analysis. Thresholds for significance were set at FC > 1.0, *p* < 0.05, and VIP > 1.0. Biological pathway analysis for differential metabolites was performed based on annotations from the KEGG database.

### 2.5. Data Statistics and Analysis

BMKCloud (www.biocloud.net, accessed on 24 January 2024) was used for sequencing data processing and statistical analysis. Prior to correlation analysis between intestinal metabolites and microbiota, all microbial OTUs were normalised by dividing the expression level of each OTU by the total expression of all OTUs within the sample. For correlation analysis between metabolites and OTUs, data were standardised using min–max normalisation. Pearson correlation analysis was then performed based on predefined filtering criteria (|CC| > 0.80 and CCP < 0.05). All statistical evaluations were performed using one-way ANOVA within the SPSS 22.0 software environment. Results are expressed as mean values accompanied by standard deviations (SD). Post hoc comparisons were made using Tukey’s HSD test to assess significance. The following thresholds were applied to denote statistical significance: * *p* < 0.05; **, *p* < 0.01; ***, *p* < 0.001.

## 3. Results

### 3.1. Diversity and Structure of the Bacterial Communities Under Heat Stress

In this study, we collected a total of 36 samples for conducting microbiome experiments, including 6 samples each for intestinal contents, intestinal mucosa, gastric contents, gastric mucosa, gill mucus, and skin mucus (with 3 HS group and 3 CO group samples in each group). Following quality filtering, a total of 262,861 high-quality sequences were generated, with individual sample counts varying between 79,477 and 80,476. After random resampling, the filtered sequences were grouped into 1855 OTUs. Good’s coverage values ranged from 99.63% to 99.76% (Table S1). Rarefaction plots levelled off, while Shannon diversity remained consistent (Figure S1), suggesting that most microbial diversity within the samples had been effectively captured.

Tukey’s HSD test was employed to assess the significance of differences in richness and diversity of microbiota across different anatomical sites of rainbow trout maintained at optimal temperature (Figure S2). The Chao1 index, based on OTU-level data, indicated relatively minor differences in microbiota richness between anatomical sites. Under suitable water temperature, the gastric mucosa exhibited significantly greater richness compared to the gastric digesta (*p* < 0.01) and also significantly higher than that of intestinal digesta and intestinal mucosa (*p* < 0.05). Simpson and Shannon diversity indices calculated at the OTU level demonstrated significant variation in microbial diversity among different anatomical locations. The diversity of microbiota in intestinal digesta, gastric mucosa, skin mucosa, and gill mucosa was higher than that observed in intestinal mucosa and gastric digesta under appropriate thermal conditions.

**Figure 1:**
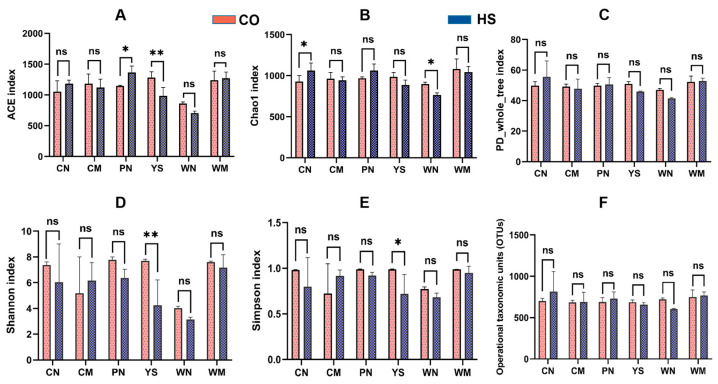
Variations in bacterial alpha diversity at different body sites under thermal stress conditions. (**A**) ACE index; (**B**) Chao-1 index; (**C**), PD_whole_tree index; (**D**) Shannon index; (**E**) Simpson index; (**F**) Operational taxonomic units (OTUs). CO, control group; HS, heat stress group; CN, intestinal digests; CM, intestinal mucosae; PN, skin mucus; YS, gill mucosae; WN, stomach digests; WM, stomach mucosae. Data are expressed as means ± SD. ns, no significance; * *p* < 0.05; ** *p* < 0.01 using a one-way analysis of variance (ANOVA) followed by Tukey’s HSD test.

Comparisons of alpha diversity metrics across sample groups were conducted using Student’s *t*-tests (Figure 1). The ACE index indicated that microbial diversity in the gill mucosa was significantly reduced under heat stress (*p* < 0.01), whereas microbial diversity in the skin mucus was significantly increased. The other types of samples showed no appreciable differences between the HS and CO groups. According to the Chao1 index, microbial diversity in the intestinal digesta was significantly increased (*p* < 0.05), while a significant decrease was observed in the gastric digesta (*p* < 0.05) under heat stress. No significant changes were found in other tissues. The PD-whole tree index showed no significant difference between the HS and CO groups. Based on the Shannon index, microbial diversity in the gill mucosa was significantly reduced under heat stress (*p* < 0.01), while other sample types exhibited no significant differences. The Simpson index also demonstrated a significant reduction in microbial diversity in the gill mucosa under heat stress (*p* < 0.05), with no significant differences in the remaining samples.

These results suggest that heat stress has a more pronounced impact on microbiota richness and diversity in the skin mucus and gill mucosa than in the digestive tract mucosa and digesta. The effect was most significant in the gill mucosa, where all five alpha diversity indices were reduced compared with the CO group. In contrast, the effects of heat stress on the intestinal mucosa, intestinal digesta, gastric mucosa, and gastric contents were relatively minor and mostly non-significant when compared with the CO group.

Multivariate statistical methods were applied to evaluate the overall composition of bacterial communities across different anatomical sites in heat-stressed rainbow trout. Results from ANOSIM testing indicated that the bacterial community structure of the gill mucosa exhibited significant alterations under heat stress (*p* = 0.001) (Table 1).

**Figure 2:**
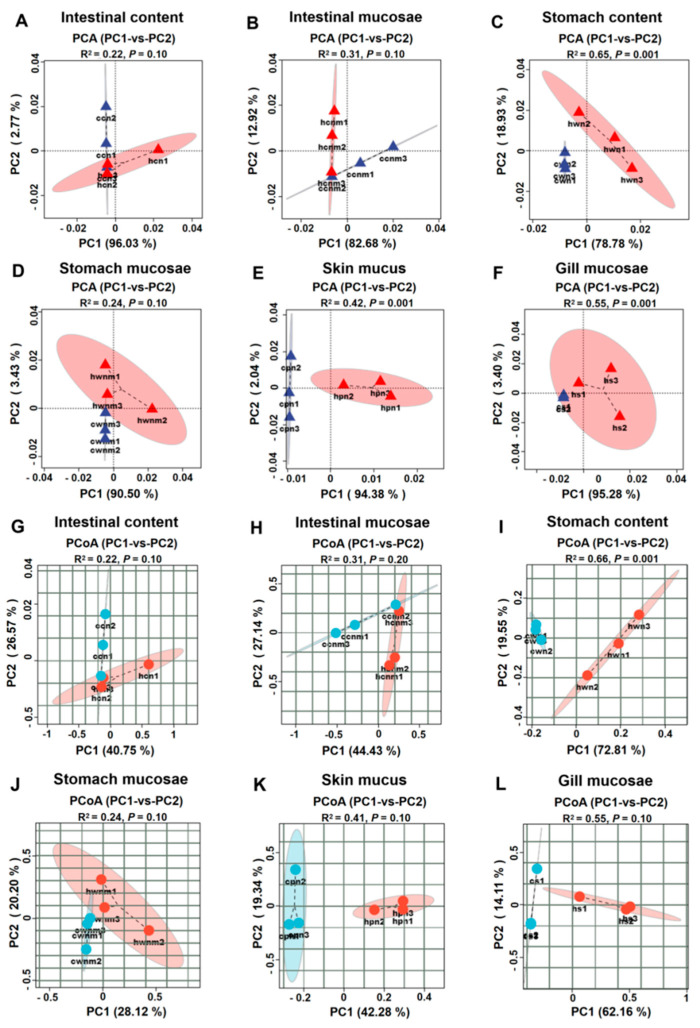
Differences in bacterial community composition across anatomical locations under thermal stress. (**A**–**F**) OTUs—based PCA plot; (**G**–**L**) unweighted Unifrac Distance-based PCoA plot; (**A**,**G**) intestinal contents; (**B**,**H**) intestinal mucosae; (**C**,**I**) stomach contents; (**D**,**J**) stomach mucosae. (**E**,**K**) skin mucus; (**F**,**L**) gill mucosae.

Sample similarity between groups was assessed using the Bray–Curtis algorithm, with distances between samples reflecting compositional differences. Principal component analysis (PCA) was used to evaluate similarity in bacterial community composition between groups (Figure 2A–F). Under thermal stress conditions, PC1 and PC2 captured 96.03% and 2.77% of the variance in intestinal digesta microbiota composition, respectively; 82.68% and 12.92% in intestinal mucosa; 78.78% and 18.93% in gastric digesta; 90.50% and 3.43% in gastric mucosa; 94.38% and 2.04% in skin mucus; and 95.28% and 3.40% in gill mucosa. These results suggest that heat stress induces compositional changes in the microbiota of the stomach, intestine, skin, and gills of rainbow trout.

The PCoA plot visualised the ANOSIM results, showing no clear separation in bacterial communities across the six anatomical sites of rainbow trout under heat stress condition (Figure 2G−L). In total, the first two principal coordinates derived from the PCoA accounted for 15.66% of the overall variation among all samples (Figure S3a). Importantly, statistical analysis revealed no significant changes in the microbial communities of the gastrointestinal digesta, gastrointestinal mucosa, or skin mucosa under heat stress (*p* > 0.05) (Table 1). However, global analysis of all samples revealed significant differences in microbial communities across the different anatomical sites of rainbow trout (*p* = 0.001), indicating that while heat stress affected microbial composition, its influence on community divergence across anatomical locations was limited (Figure S3b). Hierarchical clustering at the OTU level showed that microbial communities from different anatomical sites under normal conditions tended to cluster by site, whereas communities under heat stress did not form distinct clusters (Figure S3c).

### 3.2. Taxonomic Shifts in Bacterial Populations Triggered by Heat Stress

**Figure 3:**
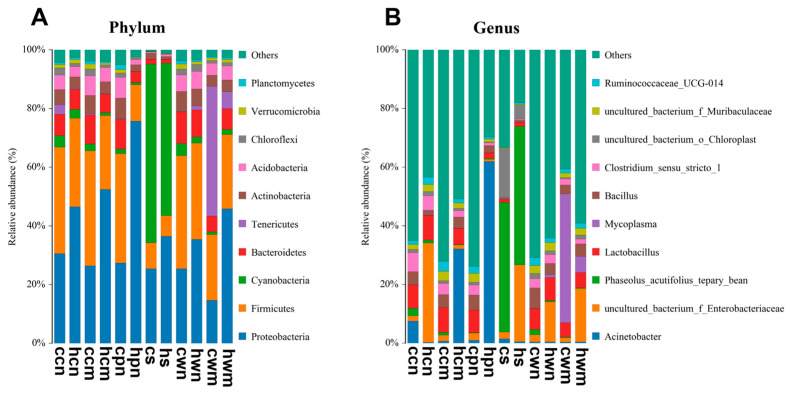
Composition and distribution of microbiota in different sites under heat stress: (**A**) at the phylum level; (**B**) at the genus level. Note: ccn, intestinal contents in control group; hcn, intestinal contents in heat stress group; cpn, skin mucosa in control group; hpn, skin mucous in heat stress group; cs, gill mucosa in control group; hs, gill mucosa in heat stress group; cwn, stomach contents in control group; hwn, stomach contents in heat stress group; cwm, stomach mucosae in control group; hwm, stomach mucosae in heat stress; ccm, intestinal mucosae in control group; hcm, intestinal mucosae in heat stress group.

To investigate how heat stress influences microbial community composition across distinct anatomical regions in rainbow trout, 16S rRNA amplicon sequencing was employed to assess the commensal microbiota at the phylum level (Figure 3A). At this taxonomic level, *Proteobacteria*, *Firmicutes*, *Cyanobacteria*, *Bacteroidetes*, *Tenericutes*, *Actinobacteria*, *Acidobacteria*, *Chloroflexi*, *Verrucomicrobia*, and *Planctomycetes* were identified as the ten most dominant phyla across the gastrointestinal tract, skin mucosa, and gill mucosa. Among these, Proteobacteria exhibited the most pronounced changes across different anatomical sites under heat stress, showing significantly higher relative abundance in the HS group compared with the CO group. With the exception of the intestinal mucosa, a decrease in Firmicutes levels was observed at various sites in fish exposed to heat stress, compared to controls. Notably, under heat stress, the relative abundance of *Cyanobacteria* in gastric contents decreased from 60.91% to 52.03%. A marked reduction in Tenericutes was observed in the intestinal mucosa, with relative abundance decreasing by 38.45%, from 44.24% to 5.79% under heat stress. In the skin mucosa, the proportion of *Proteobacteria* increased by 26.03%, while Firmicutes decreased by 14.00%. Similarly, in the gill mucosa, Proteobacteria increased by 48.35%, whereas Firmicutes decreased by 24.83% under heat stress.

Genus-level analysis of the commensal microbiota under heat stress conditions was conducted (Figure 3B). A pronounced increase in the relative abundance of Acinetobacter was observed, from 6.73% to 32.16% in the skin mucus and from 0.99% to 61.98% in the gill mucosa under heat stress. In contrast, the abundances of *Lactobacillus*, *Bacillus*, *Clostridium*, *uncultured_bacterium_f_Muribaculaceae*, *Enterobacteriaceae*, *Ralstonia*, *Ruminococcaceae_UCG-014*, *Bacteroidetes*, *Escherichia-Shigella*, and *Lysinibacillus* decreased to varying extents. Substantial compositional shifts were also observed in the intestinal digesta, intestinal mucosa, gastric digesta, and gastric mucosa under heat stress. The most notable increase was observed in *uncultured_bacterium_f_Enterobacteriaceae*, which rose from 1.78% to 33.79% in intestinal digesta, from 2.24% to 26.13% in gastric digesta, and from 2.40% to 13.59% in gastric mucosa. The relative abundance of *Mycoplasma* in the intestinal mucosa decreased significantly, from 43.97% to 5.48%. Under heat stress, the genera that decreased significantly in the intestinal digesta, intestinal mucosa, gastric digesta, and gastric mucosa included *Clostridium*, *Acinetobacter*, and *Ralstonia*, while the genera that increased significantly included *Lactobacillus*, *uncultured_bacterium_f_Muribaculaceae*, *uncultured_bacterium_f_Enterobacteriaceae*, and *Aeromonas*. These results indicate that heat stress significantly alters the composition and abundance of commensal microbiota in the gastrointestinal tract, skin mucosa, and gill mucosa of rainbow trout, and these alterations may be associated with the species’ environmental adaptability.

### 3.3. Comparative Analysis of Microbiota Across Anatomical Sites Under Heat Stress

**Figure 4:**
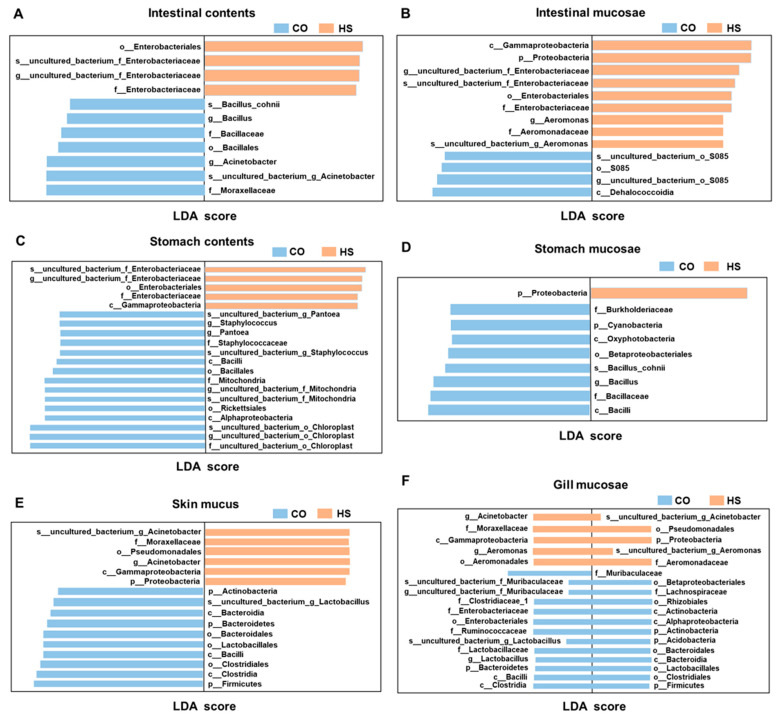
Analysis of microbiota difference in different anatomical sites under heat stress analysis of LEfSe difference: (**A**) intestinal contents; (**B**) intestinal mucosa; (**C**) stomach contents; (**D**) stomach mucosa; (**E**) skin mucus; (**F**) gill mucosae. The screening condition was LDA Score greater than 4.0. Note: CO, control group; HS, heat stress group.

LEfSe analysis was performed to detect microbial biomarkers that exhibited significant differences between groups, spanning taxonomic levels from phylum to genus (LDA score > 4.0) (Figure 4). In the intestinal digesta samples, a total of 11 taxa were found to differ significantly, comprising 2 orders, 3 families, 3 genera, and 2 species. Among these, *Bacillus*, *Moraxellaceae*, and *Acinetobacter* were identified as marker taxa in the CO group, while Enterobacteriaceae was the primary marker in the HS group. From the intestinal mucosa samples, 13 differential taxa were identified, comprising 1 phylum, 2 classes, 2 orders, 2 families, 3 genera, and 3 species. Within these, *uncultured_bacterium–S085* was the marker taxon in the CO group, while Aeromonas and Enterobacteriaceae were the markers in the HS group. In the gastric digesta, 20 differential taxa were identified, including 3 classes, 3 orders, 3 families, 6 genera, and 5 species. *Pantoea*, *Staphylococcus*, *Mitochondria*, and *Chloroplast* were marker taxa in the CO group, while Enterobacteriaceae was identified as the marker in the HS group. For gastric mucosa samples, 9 differential taxa were identified, comprising 2 phyla, 2 classes, 1 order, 2 families, 1 genus, and 1 species. *Bacillus* was the marker taxon in the CO group, whereas *Proteobacteria* was the dominant marker in the HS group. In skin mucosa samples, 16 differential taxa were identified, including 4 phyla, 4 classes, 4 orders, 1 family, 1 genus, and 2 species. *Lactobacillus* was the marker taxon in the CO group, while Proteobacteria and Acinetobacter were identified as markers in the HS group. Finally, 36 differential taxa were identified in the gill mucosa, comprising 5 phyla, 6 classes, 8 orders, 9 families, 4 genera, and 4 species. The CO group was characterised by the presence of *Muribaculaceae* and *Lactobacillus*, whereas *Aeromonas* and *Acinetobacter* were dominant in the HS group.

### 3.4. Effects of Heat Stress on Intestinal Metabolism

**Figure 5:**
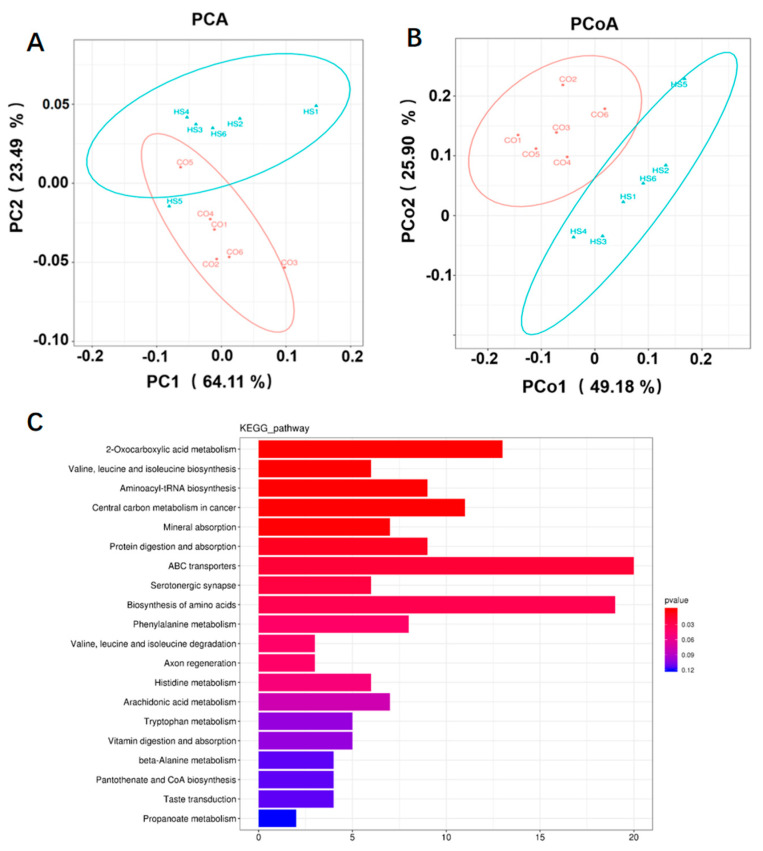
Metabolomic profile of intestinal digesta under heat stress. (**A**) PCA; (**B**) PCoA; (**C**) KEGG pathway enrichment analysis on differentially expressed metabolites in the intestinal digestive fluid under heat stress. Note: CO, control group; HS, heat stress group.

To investigate the characteristics of the intestinal metabolic profile and microbiota-derived metabolites in rainbow trout under heat stress condition, UPLC−MS analysis was conducted on 12 samples (6 from each group). As shown in Figure 5A, heat stress induced a clear separation in the metabolic profiles of intestinal contents compared to the CO group. Similarly, PCoA analysis demonstrated a distinct separation of intestinal metabolites between the two groups, indicating that heat stress significantly affected the intestinal metabolite composition in rainbow trout (Figure 5B). The OPLS−DA score plots also revealed clear separation between the CO and HS groups, confirming the alteration of metabolic profiles in the intestinal digesta under heat stress (Figure S4a,b). Analysis of differential metabolites revealed 442 significantly altered compounds between the groups, with 25 showing increased levels and 417 showing decreased levels in the HS group relative to the CO group (Figure S4c). To gain deeper insights into the metabolic adaptations triggered by thermal stress, pathway enrichment analysis was performed on metabolites from the intestinal digesta. A total of 1170 metabolites were mapped to enriched pathways, which included 2−oxocarboxylic acid metabolism; biosynthesis of valine, leucine, and isoleucine; aminoacyl−tRNA biosynthesis; central carbon metabolism in cancer; mineral and nutrient absorption; protein digestion and absorption; ABC transporter systems; serotonergic synapse signalling; amino acid biosynthesis and degradation; axon regeneration; histidine, arachidonic acid, and tryptophan metabolism; vitamin absorption; beta−alanine metabolism; pantothenate and CoA biosynthesis; taste transduction; and propanoate metabolism (Figure 5C). Functional enrichment analysis of intestinal metabolites revealed significant enrichment across multiple metabolic pathways, particularly in histidine metabolism, arachidonic acid metabolism, tryptophan metabolism, and beta−alanine metabolism. Collectively, these findings suggest that heat stress significantly alters intestinal metabolic processes in rainbow trout.

### 3.5. Intestinal Metabolomic Landscape and Its Association with Gut Microbiota

**Figure 6:**
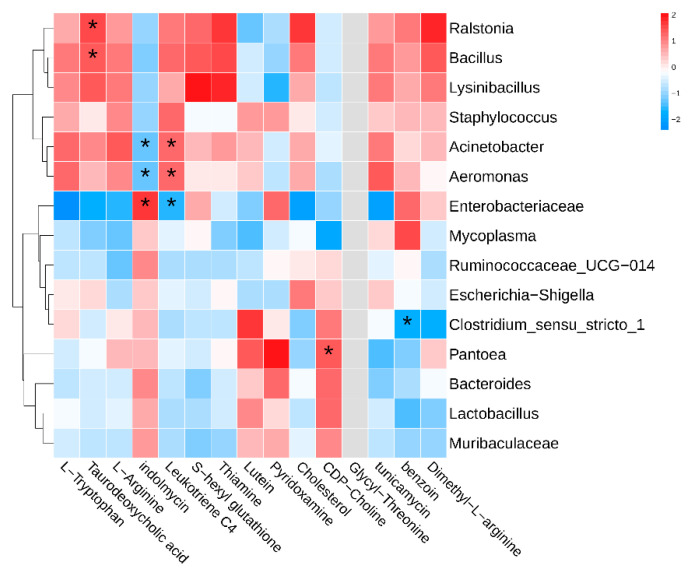
Correlation patterns between intestinal microbial taxa and metabolites in rainbow trout subjected to heat stress. Red shading denotes positive correlations, while blue indicates negative associations. Asterisks (*) represent statistically significant correlations (*p* < 0.05).

In order to assess the interaction between intestinal microbiota and metabolite profiles under thermal stress in rainbow trout, Spearman’s correlation analysis was performed (Figure 6). The results revealed 10 significant associations between five intestinal metabolites and gut microbial taxa, comprising six positive correlations and four negative correlations. Taurodeoxycholic acid exhibited strong positive associations with *Ralstonia* and *Bacillus*, showing correlation coefficients of 0.943 and 0.886, respectively. *Indoleomycin* was positively linked to *Enterobacteriaceae*, while displaying negative correlations with *Aeromonas* and *Acinetobacter*, all with correlation coefficients of ±0.886. *Leukotriene C4* exhibited positive correlations with *Aeromonas* and *Acinetobacter*, and a negative correlation with *Enterobacteriaceae*, with correlation coefficients of 0.886, 0.886, and −0.886, respectively. *CDP*−*Choline* was positively correlated with *Pantoea* (r = 0.943), while *Benzoin* showed a negative correlation with *Clostridium_sensu_stricto_1* (r = −0.943). These findings indicate a clear interdependent relationship between specific intestinal metabolites and microbial taxa in rainbow trout under heat stress condition.

## 4. Discussion

The fish microbiota is a complex consortium comprising protists, yeasts, viruses, bacteria, and archaea [18]. These microbial communities inhabit both external mucosal surfaces (such as the skin and gills) and internal mucosal environments (including the gastrointestinal tract and digesta), and their abundance and composition are shaped by multiple factors, including temperature, seasonality, host genetics, diet, and pathogens [19,20]. It has been shown that the phylogenetic composition of the fish gut microbiome is shaped by both species-specific factors—such as host lineage, genetic background, and dietary habits—and environmental conditions, including water chemistry and the makeup of planktonic bacterial communities. Species-specific factors predominantly influence the gut microbiota, whereas the skin mucus microbiota is more closely associated with environmental physicochemical parameters and the structure of planktonic microbial communities [21]. The composition and abundance of microorganisms vary substantially across fish tissues (skin, gills, intestines) and digesta (faeces). However, limited information is available regarding the changes in commensal microbiota under heat stress and their relationship to host environmental adaptability. We investigated the alterations in microbiota within internal sites (intestinal digesta, intestinal mucosa, gastric digesta, and gastric mucosa) and surface mucosa (skin and gill) of rainbow trout under heat stress condition and examined the site-specific microbial response mechanisms to thermal stress.

### 4.1. Disruption of Commensal Microbiota Under Heat Stress Conditions

The skin and gills form the outer mucosal barrier of fish and play a critical role in protection against pathogens and toxins. Most microorganisms residing in these sites are obligate aerobes and serve as potential biomarkers of fish health [22]. Compared with intestinal commensal bacteria, skin mucus bacteria are more tolerant of harsh environmental conditions and contribute significantly to defence against opportunistic pathogens through colonisation resistance mechanisms [21]. Typically, *Proteobacteria* dominate the skin mucus microbiota, while *Bacteroidetes*, *Actinobacteria*, *Firmicutes*, and *Verrucomicrobia* are present in relatively lower abundance [18,23,24,25]. Previous studies have reported that *Proteobacteria*, *Firmicutes*, and *Actinobacteria* are the most common commensal bacteria found in both gills and skin [26,27,28]. Lowrey et al. [29] observed that microbial composition differs between the gill and skin of rainbow trout; *Firmicutes* and *Actinobacteria* were more prevalent in the skin mucosa, whereas *Proteobacteria* and *Bacteroides* dominated in the gills, potentially reflecting their role in facilitating gas exchange.

In the present study, the microbial composition of skin and gill mucosa in rainbow trout under heat stress condition was similar, with *Proteobacteria*, *Firmicutes*, and *Bacteroidetes* constituting the dominant phyla in both tissues. The most pronounced change under heat stress was an increase in the relative abundance of *Proteobacteria*, which was significantly higher in the HS group than in the CO group. Conversely, the proportion of *Firmicutes* was significantly lower in the HS group. Notably, the relative abundance of Acinetobacter increased from 6.73% to 32.16% in the skin mucus and from 0.99% to 61.98% in the gill mucosa under heat stress. The observed increase in *Proteobacteria* and *Acinetobacter*, along with the decrease in *Firmicutes*, suggests that heat stress may disrupt the microbial balance in the skin and gill mucosa of rainbow trout, potentially leading to dysbiosis and compromising host health.

The stomach and intestine are key components of the fish digestive system, and their associated microbial communities play an essential role in maintaining host homeostasis and environmental adaptability [30]. In the present study, heat stress exerted the most pronounced effects on the microbial diversity of the gill mucosa and skin mucus in rainbow trout, followed by the gastric and intestinal digesta. In contrast, the microbial diversity of the gastric and intestinal mucosa was relatively unaffected. Hao et al. [31] found that the composition of microbiota in different anatomical sites of Microcephalus is primarily influenced by environmental factors, with the highest microbial diversity observed in the skin mucosa, and comparatively lower diversity in the intestinal mucosa and digesta. Other studies have shown that environmental temperature changes can significantly alter the composition and richness of host-associated microbial communities in animals, potentially exerting adverse effects on the host, as observed in the Chinese giant salamander [4], red-backed salamander [5], and common lizard [6].

Significant differences in the species composition and relative abundance of dominant gastric and intestinal microbial communities have been observed in fish under varying environmental conditions. Previous studies have shown that *Proteobacteria* is the most dominant gut microbiota in most marine and freshwater fish, followed by Firmicutes, *Fusobacteria*, *Bacteroidetes*, *Actinobacteria*, and *Tenericutes* [32]. In the present study, *Proteobacteria*, *Firmicutes*, and *Bacteroidetes* were the three dominant phyla in the gastrointestinal digesta and surface mucosa of rainbow trout under heat stress condition, which is generally consistent with previous findings. In terms of microbial composition, *Cyanobacteria* accounted for more than 60% of the microbial community in the stomach, showing a distinct profile compared to other anatomical sites. Under heat stress, *Proteobacteria* exhibited the most significant increase in relative abundance in both intestinal digesta and mucosa, with levels significantly higher in the HS group compared to the CO group. Except for the intestinal mucosa, the relative abundance of Firmicutes was lower in the HS group than in the CO group. Exposure to different osmotic pressures (freshwater vs. seawater) has also been shown to alter the microbial composition of the skin, mucosa, and gut in Atlantic salmon. Notably, the transition from freshwater to seawater resulted in an increased abundance of Proteobacteria and a decreased abundance of *Bacteroidetes* in the skin mucus [33]. In the intestine, *Actinobacteria* and *Proteobacteria* were the most affected, while *Firmicutes* showed a significant increase [34]. These results suggest that the composition and structure of fish microbiota respond differently to various environmental stressors.

An imbalance in the taxonomic composition of the microbiota is referred to as dysbiosis. The surface mucosa of fish is in direct and continuous contact with the aquatic environment, and its homeostasis is constantly influenced by external factors. Temperature is a primary environmental stressor in fish, often disrupting the balance between host-associated microbiota and pathogens. Disruption of the mucosal microbiota may lead to disease and mortality in fish [19]. Studies have shown that environmental changes can alter the mucosal microbiota, facilitating the colonisation of opportunistic pathogens on mucosal surfaces [35,36]. An increased abundance of *Proteobacteria* is recognised as a microbial hallmark of intestinal dysbiosis [37]. First proposed by Stackebrandt et al. in 1988 [38], *Proteobacteria* are characterised by the presence of lipopolysaccharide in their outer membrane and a Gram-negative cell wall structure. They represent the largest known bacterial phylum [37]. Proteobacteria have been implicated in the disturbance of intestinal equilibrium, with their increased presence in the gut strongly linked to inflammatory bowel disorders, including Crohn’s disease (CD) and ulcerative colitis (UC) [37]. However, the precise mechanisms driving the proliferation of *Proteobacteria* during IBD remain unclear. In our previous study, heat stress induced intestinal injury in rainbow trout, resulting in increased abundance of Proteobacteria and expansion of *Enterobacteriaceae* (facultative anaerobes), accompanied by a reduction in obligate anaerobes, thereby promoting gut dysbiosis [39]. *Bacteroides* are among the most common members of the intestinal microbiota and play a key regulatory role in intestinal health. While some *Bacteroides* species are pathogenic, most are commensal and colonise the intestinal mucosa, forming symbiotic relationships with the host and inhibiting the colonisation of intestinal pathogens [17]. *Bacteroidetes* are involved in carbohydrate transport and protein metabolism and contribute significantly to digestive efficiency in animals [40]. Thus, the observed decrease in the abundance of *Bacteroidetes* under heat stress may impair the normal digestive and absorptive functions of rainbow trout. The decline in *Bacteroidetes* and *Firmicutes* observed under thermal stress may disrupt feeding efficiency, digestion, and nutrient uptake in rainbow trout. The *Firmicutes-to-Bacteroidetes* ratio is commonly used as an indicator of gut physiological status, with a decline in *Firmicutes* associated with intestinal dysfunction [41,42]. *Firmicutes* are considered key butyrate-producing bacteria [43], supplying energy to intestinal mucosal cells, maintaining mucosal integrity, and supporting the intestinal microecological balance [44,45]. Under acute heat stress, *Firmicutes* levels in the intestinal digesta of rainbow trout is significantly reduced [39], suggesting that disruption of the intestinal mucosal barrier and microbial imbalance may be linked to changes in *Firmicutes* abundance and short-chain fatty acid (SCFA) production [44,46].

The commensal microbiota at the genus level exhibited site-specific variation in rainbow trout under heat stress condition. In the present study, microbial compositional changes in the skin mucus and gill mucosa under heat stress were notably similar. The relative abundance of Acinetobacter increased significantly from 6.73% to 32.16% in skin mucus and from 0.99% to 61.98% in gill mucosa. Acinetobacter is recognised as a serious human pathogen, implicated in conditions such as bacteraemia, pneumonia, and meningitis. However, reports on the role of Acinetobacter in fish-associated microbiota remain limited. Cao et al. [47] first confirmed its pathogenicity in fish through challenge experiments in Schizothorax prenanti, Schizothorax davidi, and Schizothorax wangchiachii. Boutin et al. [48] observed that hypoxic stress reduced the abundance of mucosal probiotics and increased the prevalence of pathogenic taxa, including *Coldbacilli*, *Steroidobacteria*, *Pseudomonas*, *Acinetobacter*, and *Aeromonas*, in the skin mucus of *Salvelinus* fontinalis. The primary genera of pathogenic bacteria associated with the mucosal surfaces of teleost fish include *Pseudomonas*, *Vibrio*, *Nephrobacterium*, *Luminobacter*, *Edwardsiella*, *Lactococcus*, *Pasteurella*, *Streptococcus*, *Flavobacterium*, *Tenacibaculum*, *Mycobacterium*, and *Yersinia* [49]. Under healthy physiological conditions, these potential pathogens typically do not pose a threat to the host. However, when fish experience physiological imbalance due to elevated environmental temperature, the host becomes more susceptible to infection. Under heat stress, suppression of the innate immune system may permit environmental pathogens (such as parasites) to secrete immunosuppressive compounds that degrade the mucus layer, which serves as the primary barrier preventing pathogen invasion in teleost fish [50,51].

In the present study, *Enterobacteriaceae* exhibited the most pronounced compositional changes among gastrointestinal microbial communities under heat stress. Its relative abundance increased from 1.78% to 33.79% in intestinal digesta, from 1.44% to 18.16% in intestinal mucosa, from 2.24% to 26.13% in gastric digesta, and from 2.40% to 13.59% in gastric mucosa. Enterobacteriaceae is a family within the order *Enterobacterales* of the class *Gammaproteobacteria*, comprising Gram-negative bacteria commonly found in the intestines of animals, as well as in water, soil, and other environments. Members of this family include opportunistic pathogens such as *Escherichia*, *Morganella*, and *Pantobacteroides*, as well as established pathogenic genera such as *Salmonella*, *Shigella*, and *Yersinia*. Previous studies have reported a significant increase in the abundance of Enterobacteriaceae during the onset and progression of intestinal diseases [52]. Inflammatory responses initiated by the host have been reported to alter the gut microbial balance and facilitate the proliferation of *Enterobacteriaceae* [52]. Accordingly, the marked increase in *Enterobacteriaceae* observed in both surface and internal microbiota of rainbow trout under heat stress condition suggests that elevated temperatures may disturb the balance of symbiotic microbiota and have detrimental effects on fish health and aquaculture viability.

In addition, the relative abundance of *Mycoplasma* in the intestinal mucosa decreased markedly, from 43.97% to 5.48%. Limited research is available on *Mycoplasma* in fish, and its role in host health remains poorly defined. A study on the intestinal microbiota of Atlantic salmon reported that *Mycoplasma* was dominant in healthy individuals, whereas its abundance was notably reduced in diseased fish [53], which aligns with the results observed in this study. Ofek et al. [54] similarly reported high variability in the abundance of *Mycoplasma* in the gut and spleen microbiota of healthy and diseased *Tilapia*. *Mycoplasma* has also been identified as a dominant genus in rainbow trout, accounting for up to 83% of the gut microbiota [8,55] and even up to 92% in Atlantic salmon [23,37,56]. In addition to its potential association with nutrient production and weight gain, *Mycoplasma* has also been linked to disease and tumour development in fish [53]. While its precise role remains unclear, some evidence suggests that *Mycoplasma* may participate in energy metabolism. For example, an increased abundance of *Mycoplasma* has been observed in Atlantic salmon fed a high-fat diet, potentially contributing to weight gain [57]. Moreover, *Mycoplasma* has been implicated in the development of intestinal infectious tumours in aged zebrafish and is believed to play a role in tumour occurrence and transmission [58]. Therefore, the significant reduction in *Mycoplasma* abundance observed in the intestinal mucosa of rainbow trout under heat stress condition may impair their environmental adaptability.

### 4.2. Association Analysis of Gut Microbiota and Its Metabolites Under Heat Stress Condition

Gut microbial metabolism plays a critical role in sustaining host physiological balance and general well-being. Alterations in the composition and abundance of gut microbiota under extreme environmental conditions can lead to shifts in intestinal metabolites, thereby influencing host phenotypes [14]. Previous studies have suggested that reduced productivity in heat-stressed animals is often attributed to decreased nutrient intake [15]. However, Baumgard et al. [59] reported that heat stress alters physiological metabolic processes through regulatory mechanisms involving metabolism and preferential energy allocation, independent of nutrient intake or energy balance. Such stress-induced physiological changes affect the metabolism of carbohydrates, lipids, and proteins via coordinated adjustments in energy supply and utilisation across multiple organs.

As the core components of proteins, amino acids are intimately involved in key physiological functions and metabolic activities. Amino acids, the fundamental units of proteins, are closely associated with essential life processes in organisms [17]. In the present study, the altered metabolites detected in heat-stressed rainbow trout were predominantly associated with metabolic pathways involved in protein digestion and nutrient assimilation, amino acid biosynthesis, biosynthesis and degradation of valine, leucine, and isoleucine, aminoacyl-tRNA biosynthesis, phenylalanine metabolism, histidine metabolism, and tryptophan metabolism. Earlier research has indicated that disruptions in amino acid metabolism may lead to compromised immune function and elevated oxidative stress, which can, in turn, hinder muscle growth and contribute to weight loss [60,61]. Aminoacyl-tRNA has been implicated in key physiological and pathological processes, including inflammation, immune responses, and tumour development [62,63]. This implies that the intestinal damage and inflammation observed in heat-stressed rainbow trout could be linked to impairments in the aminoacyl-tRNA biosynthesis pathway.

Alterations in microbial species composition can influence amino acid digestion and absorption in animals [64]. Our findings showed that most altered intestinal metabolites under heat stress in rainbow trout were concentrated in pathways regulating amino acid metabolism, particularly those related to protein digestion, absorption, and amino acid biosynthesis. For example, *Clostridium* in the animal gut has been identified as a key driver of host amino acid fermentation, while *Peptostreptococcus* plays a central role in glutamate and tryptophan utilisation [65]. Tryptophan is an essential aromatic amino acid, structurally characterised by a β-carbon attached to the third position of an indole ring [66]. In the gut, tryptophan metabolism involves its microbial conversion into various indole derivatives, which function as important modulators and inhibitors of inflammation, contributing to immune responses at epithelial barriers and supporting mucosal integrity [66,67,68]. In our previous study, acute thermal stress in rainbow trout was associated with an inverse relationship between serum tryptophan levels and the populations of certain intestinal microbes, including *Bacteroides* and *Enterobacter* [39]. Furthermore, tryptophan metabolism in the gut is directly or indirectly regulated by microbiota across multiple physiological pathways [66]. From an aquaculture perspective, gut tryptophan metabolism represents a potentially modifiable factor. Dietary supplementation or the use of probiotics to regulate microbial taxa involved in tryptophan metabolism may offer a promising strategy to enhance stress resilience in fish.

## 5. Conclusions

In summary, this study provides a comprehensive taxonomic and functional analysis of the microbial composition and diversity present in the skin mucus, gill mucosa, gastrointestinal digesta, and gastrointestinal mucosa, as well as the metabolic profile of intestinal digesta in rainbow trout under heat stress conditions. Microbial composition and diversity analyses revealed that heat stress had the most pronounced effects on the gill and skin mucus microbiota, followed by the gastrointestinal digesta, while relatively minor effects were observed in the gastrointestinal mucosa. *Proteobacteria*, *Firmicutes*, and *Bacteroidetes* exhibited the most notable compositional shifts in the intestinal digesta, gastrointestinal mucosa, and surface mucosa under heat stress. *Cyanobacteria* remained the dominant microbial group in the stomach. Heat stress led to a marked increase in the relative abundance of *Acinetobacter* in the skin mucus and gill mucosa as well as Enterobacteriaceae in the intestinal digesta, intestinal mucosa, gastric digesta, and gastric mucosa. Additionally, heat stress altered the composition of intestinal microbiota-associated metabolites. Most of the altered metabolites identified in the digesta were significantly associated with metabolic pathways involved in amino acid processing, particularly tryptophan metabolism. Despite these findings, further research is needed to clarify the adaptive mechanisms of fish commensal microbiota and intestinal metabolites in response to elevated water temperatures.

## Figures and Tables

**Table 1:** Overall structural differences in bacterial communities across different anatomical sites of rainbow trout under heat stress condition.

Group	Community Structure	
	R^2^	*p*
Whole comparison	0.663	0.001
ccn vs. hcn	0.202	0.301
cpn vs. hpn	0.218	0.200
cs vs. hs	0.242	0.001
cwn vs. hwn	0.264	0.100
cwm vs. hwm	0.207	0.500
ccm vs. hcm	0.204	0.301

Note: ccn, intestinal contents in control group; hcn, intestinal contents in heat stress group; cpn, skin mucosa in control group; hpn, skin mucous in heat stress group; cs, gill mucosa in control group; hs, gill mucosa in heat stress group; cwn, stomach contents in control group; hwn, stomach contents in heat stress group; cwm, stomach mucosae in control group; hwm, stomach mucosae in heat stress; ccm, intestinal mucosae in control group; hcm, intestinal mucosae in heat stress group.

## Data Availability

Data are contained within the article and Supplementary Materials.

## Supplementary Materials

The following supporting information can be downloaded at: https://www.mdpi.com/article/10.3390/ani15142017/s1, Figure S1: Rarefaction analysis of OTUs clustered at 97% sequence identity of all thirty-six samples. (a) Rarefaction curves on OTU level of all samples; (b) Shannon curves on OTU level of all samples. Note: ccn, intestinal contents of CO group; hcn, intestinal contents of HS group; ccm, intestinal mucosae of CO group; hcm, intestinal mucosae of HS group; cpn, skin mucus of CO group; hpn, skin mucus of HS group; cs, gill mucosae of CO group; hs, gill mucosae of HS group; cwn, stomach contents of CO group; hwn, stomach contents of HS group; cwm, stomach mucosae of CO group; hwm, stomach mucosae of HS group; Figure S2: Comparison of alpha diversity of the bacterial communities at different sites. (a), (b), (c): Chao-1, Simpson, and Shannon indices of different anatomical sites of rainbow trout at suitable water temperature (16 °C), respectively. Note: ccn, intestinal content; ccm, intestinal mucosae; cpn, skin mucus; cs, gill mucosae; cwn, stomach content; cwm, stomach mucosae. *, *p* < 0.05; **, *p* < 0.01; Figure S3: Analysis of the bacterial community structure at different sites. A. Principal coordinate analysis based on the Bray—Curtis metric of the bacterial communities. The percentages indicate the relative contribution of the principal components. B. ANOSIM analysis based on the Bray—Curtis metric of the bacterial communities. C. The hierarchical clustering tree based on Bray—Curtis metric of the bacterial communities. Note: ccn, intestinal digests of CO group; hcn, intestinal digests of HS group; ccm, intestinal mucosae of CO group; hcm, intestinal mucosae of HS group; cpn, skin mucus of CO group; hpn, skin mucus of HS group; cs, gill mucosae of CO group; hs, gill mucosae of HS group; cwn, stomach digests of CO group; hwn, stomach digests of HS group; cwm, stomach mucosae of CO group; hwm, stomach mucosae of HS group; Figure S4: Metabolome profile of intestinal digests under heat stress. (a) OPLS-DA model score plot; (b) Validation plot of the OPLS-DA model. (c) Volcano plot of differential metabolites. Note: CO, control group; HS, heat stress group. Table S1: Summary on raw data processing.
