# Supplementary material for: Analysis of the Alterations in Symbiotic Microbiota and Their Correlation with Intestinal Metabolites in Rainbow Trout (Oncorhynchus mykiss) Under Heat Stress Conditions
Source: Animals (Basel). 2025 Jul 8;15(14):2017. doi: 10.3390/ani15142017 (PMC12291889; doi:10.3390/ani15142017)
Supplement: Supplementary file 1 [file animals-15-02017-s001.zip › animals-3689851-supplementary.docx]

**Supplementary material**

**Table S1.** Summary on raw data processing.

| **Sample (n = 3)** | | **Raw Reads** | **Clean Reads** | **Effective Reads** | **GC(%)** | **Q20(%)** | **Q30(%)** | **Effective(%)** |
| --- | --- | --- | --- | --- | --- | --- | --- | --- |
| CN | CO | 79899 | 79680 | 75349 | 53.86 | 99.24 | 96.61 | 94.31 |
|  | HS | 79845 | 79616 | 74667 | 54.36 | 99.25 | 96.63 | 93.51 |
| CM | CO | 79924 | 79704 | 75814 | 50.37 | 99.14 | 96.18 | 94.86 |
|  | HS | 80076 | 79867 | 76414 | 53.89 | 99.23 | 96.57 | 95.42 |
| WN | CO | 80143 | 79889 | 77156 | 54.42 | 99.19 | 96.48 | 96.28 |
|  | HS | 79883 | 79650 | 77568 | 54.81 | 99.24 | 96.62 | 97.10 |
| WM | CO | 80118 | 79873 | 73726 | 54.04 | 99.20 | 96.54 | 92.02 |
|  | HS | 80008 | 79785 | 79914 | 54.30 | 99.22 | 96.57 | 92.38 |
| PN | CO | 79895 | 79669 | 74972 | 54.11 | 99.23 | 96.59 | 93.84 |
|  | HS | 79963 | 79745 | 75214 | 53.43 | 99.22 | 96.52 | 94.06 |
| YS | CO | 80024 | 79791 | 74671 | 54.32 | 99.22 | 96.56 | 93.31 |
|  | HS | 79970 | 79736 | 73964 | 52.61 | 99.21 | 96.47 | 92.49 |

CN, intestinal contents; CM, intestinal mucosae; PN, skin mucus; YS, gill mucosae; WN, stomach contents; WM, stomach mucosae; CO, control groups; HS, heat stress groups.


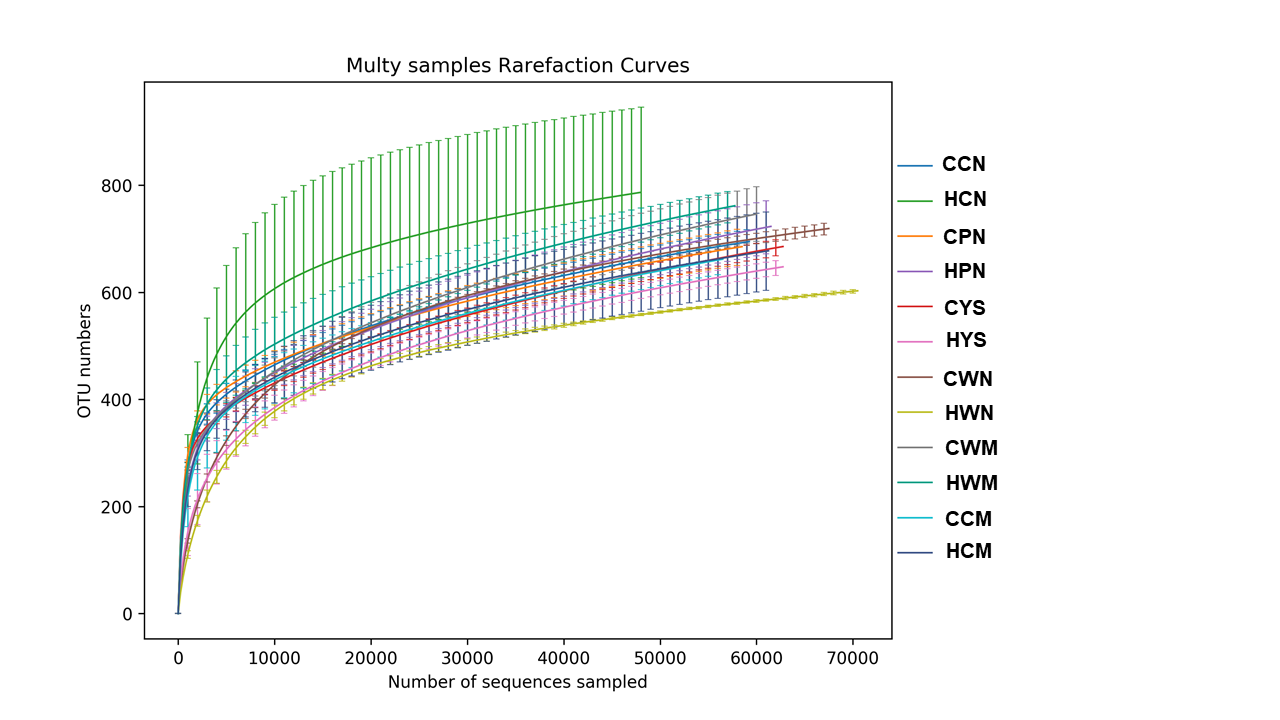


（a）


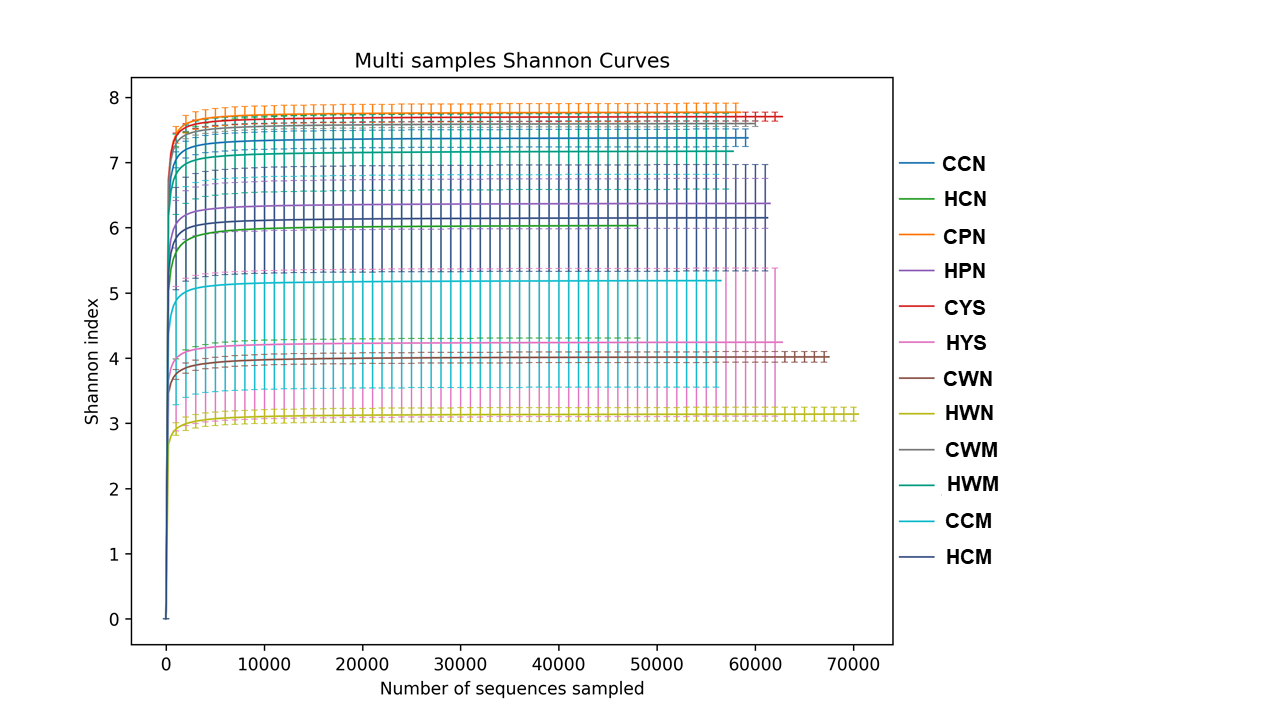


(b)

**Figure S1**. Rarefaction analysis of OTUs clustered at 97% sequence identity of all thirty-six samples. (a) Rarefaction curves on OTU level of all samples; (b) Shannon curves on OTU level of all samples. ccn, intestinal contents of CO group; hcn, intestinal contents of HS group; ccm, intestinal mucosae of CO group; hcm, intestinal mucosae of HS group; cpn, skin mucus of CO group; hpn, skin mucus of HS group; cs, gill mucosae of CO group; hs, gill mucosae of HS group; cwn, stomach contents of CO group; hwn, stomach contents of HS group; cwm, stomach mucosae of CO group; hwm, stomach mucosae of HS group.


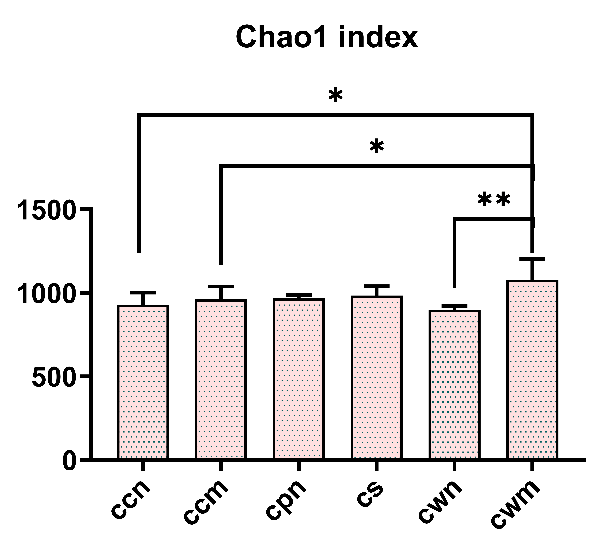

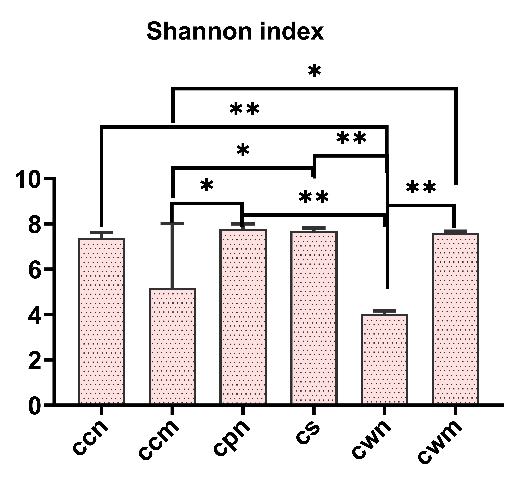


(a) (b)


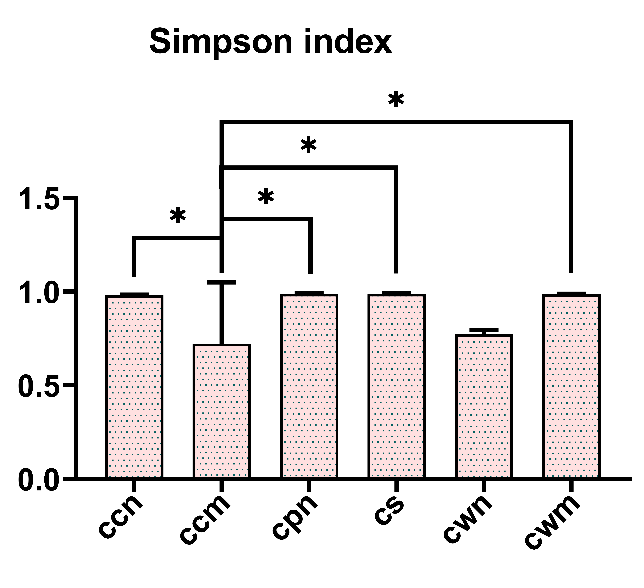


(c)

**Figure S2.** Comparison of alpha diversity of the bacterial communities at different sites. (a), (b), (c): Chao-1, Simpson, and Shannon indices of different anatomical sites of rainbow trout at suitable water temperature (16 ℃), respectively. ccn, intestinal content; ccm, intestinal mucosae; cpn, skin mucus; cs, gill mucosae; cwn, stomach content; cwm, stomach mucosae. *, *P* < 0.05; **, *P* < 0.01.


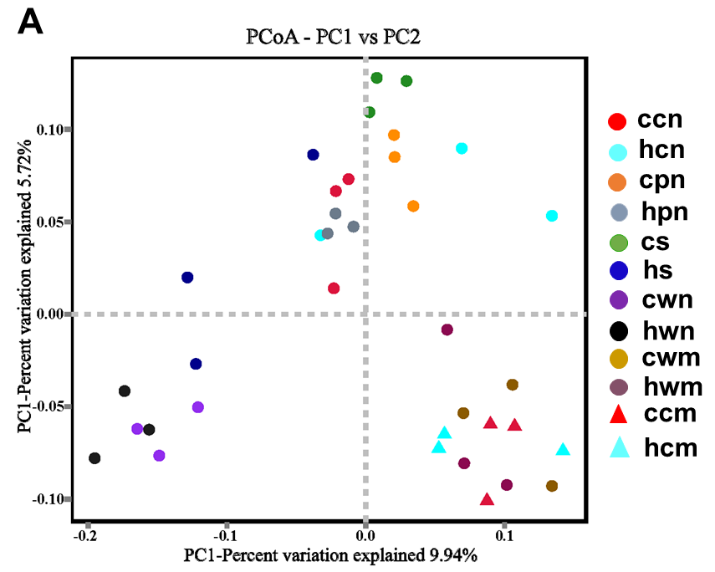


(a)


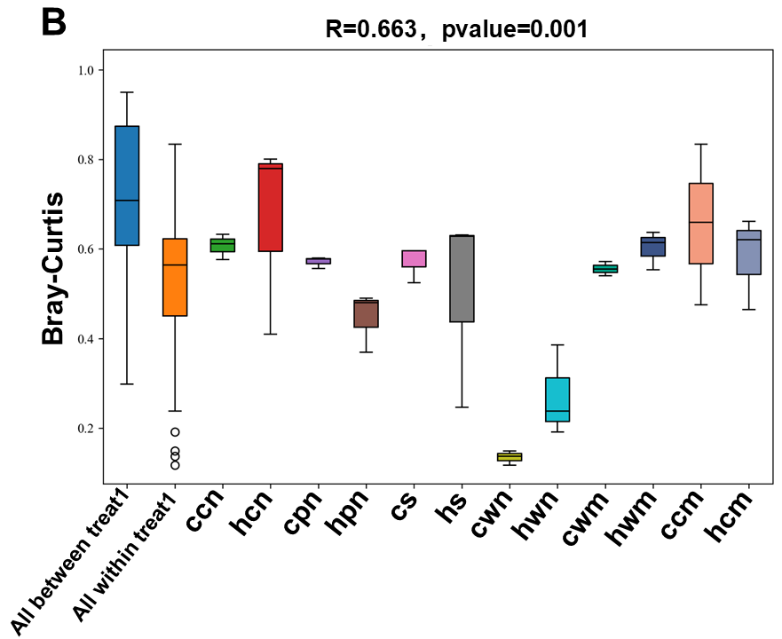


(b)


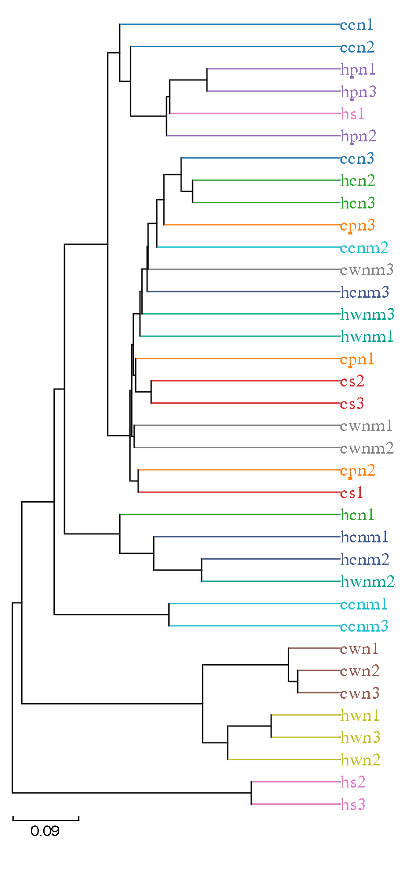


(c)

**Figure S3.** Analysis of the bacterial community structure at different sites**.** A. Principal coordinate analysis based on the Bray-Curtis metric of the bacterial communities. The percentages indicate the relative contribution of the principal components. B. ANOSIM analysis based on the Bray-Curtis metric of the bacterial communities. C. The hierarchical clustering tree based on Bray-Curtis metric of the bacterial communities. ccn, intestinal digests of CO group; hcn, intestinal digests of HS group; ccm, intestinal mucosae of CO group; hcm, intestinal mucosae of HS group; cpn, skin mucus of CO group; hpn, skin mucus of HS group; cs, gill mucosae of CO group; hs, gill mucosae of HS group; cwn, stomach digests of CO group; hwn, stomach digests of HS group; cwm, stomach mucosae of CO group; hwm, stomach mucosae of HS group.


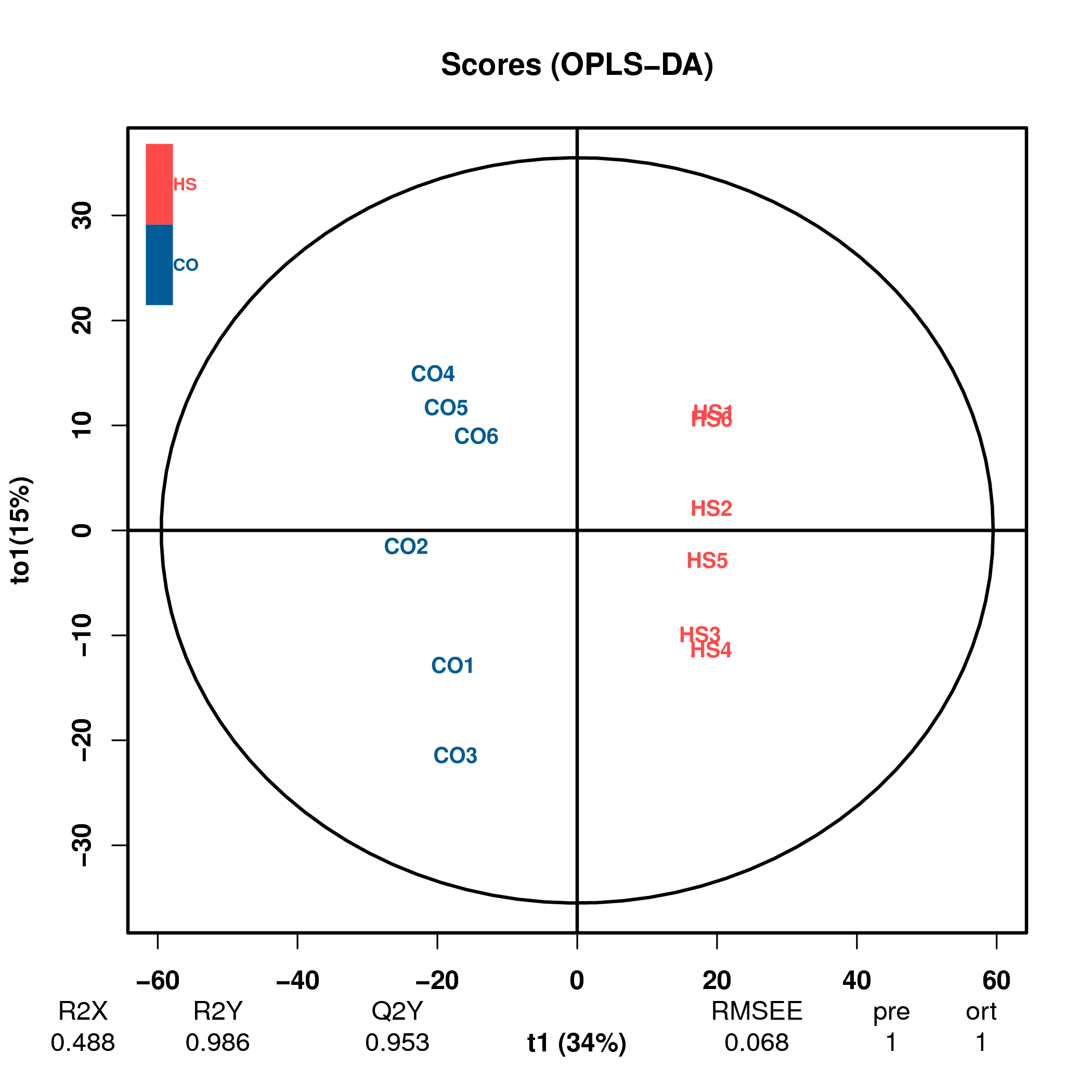


(a)


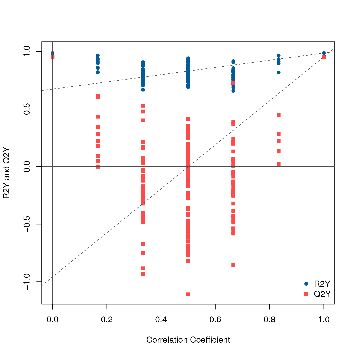


(b)


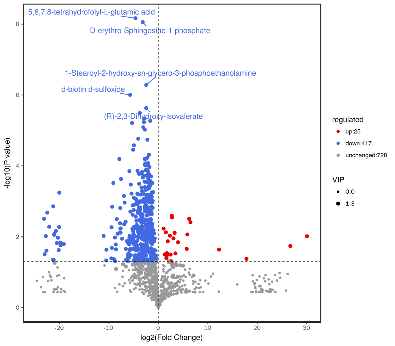


(c)

**Figure S4.** Metabolome profile of intestinal digests under heat stress**.** (a). OPLS-DA model score plot; (b). Validation plot of the OPLS-DA model. (c). Volcano plot of differential metabolites; CO, control group; HS, heat stress group.
